# Role of miR-181 Family Members in Stroke: Insights into Mechanisms and Therapeutic Potential

**DOI:** 10.3390/ijms26020440

**Published:** 2025-01-07

**Authors:** Cornelia Braicu, Fior Dafin Mureșanu, Ekaterina Isachesku, Natan Bornstein, Saša R. Filipović, Stefan Strilciuc, Adrian Pana

**Affiliations:** 1 Department of Genomics, MEDFUTURE Institute for Biomedical Research, Iuliu Hațieganu University of Medicine and Pharmacy, 400337 Cluj-Napoca, Romania; cornelia.braicu@umfcluj.ro (C.B.); ekaterina.isachesku@umfcluj.ro (E.I.); 2 RoNeuro Institute for Neurological Research and Diagnostics, 37 Mircea Eliade St., 400364 Cluj-Napoca, Romania; dafinm@ssnn.ro; 3 Department of Neuroscience, Iuliu Haţieganu University of Medicine and Pharmacy, 8 Victor Babes St., 400347 Cluj-Napoca, Romania; 4 Department of Neurology, Shaare Zedek Medical Center and Faculty of Medicine, Hebrew University of Jerusalem, Shmuel Bait 12, Jerusalem 9103102, Israel; natan.bornstein@gmail.com; 5 Institute of Psychology, Department of Psychology, Faculty of Philosophy, University of Belgrade, 11000 Belgrade, Serbia; sasa.filipovic@imi.bg.ac.rs; 6 Center for Health Outcomes & Evaluation, Splaiul Unirii 45, 030126 Bucharest, Romania; adrian.pana@me.com

**Keywords:** cerebral ischemia, miR-181 family members, biological processes

## Abstract

Stroke is a major cause of mortality and long-term disability worldwide, making early diagnosis and effective treatment crucial for reducing its impact. In response to the limited efficacy of current treatments, alternative therapeutic strategies, such as novel biomarkers and therapies, are emerging to address this critical unmet medical need. MicroRNAs (miRNAs) are small, non-coding RNAs that regulate gene expression at the post-transcriptional level. Due to their dysregulation, they have been implicated in the onset and progression of various diseases. Recent research highlighted the important role of miR-181 family members in the context of stroke. Polymorphisms such as rs322931 in miR-181b are associated with increased stroke risk. miR-181 family members are aberrantly expressed and related to various aspects of stroke pathology, affecting inflammatory responses or neuronal survival. We provide a comprehensive overview of how alterations in miR-181 expression influence stroke mechanisms and their potential as therapeutic targets.

## 1. Introduction

Cerebral ischemia occurs when blood flow to a specific brain region is interrupted, resulting from either a hemorrhagic break or an ischemic occlusion, where a blockage prevents blood from reaching brain tissue. Hemorrhagic strokes, although less common, account for approximately 15% of all stroke cases, while ischemic strokes are far more prevalent, comprising about 85% of cases [1,2]. This demonstrates the critical need for biomarkers, prevention, and treatment strategies for both types of strokes [3].

The progression of stroke is typically divided into two distinct phases: acute and chronic. The acute phase is characterized by rapid neuronal injury, which occurs within minutes to hours of the onset of ischemia. During this phase, brain cells become deprived of oxygen and nutrients, leading to cell death and the release of neurotoxic mediators that can exacerbate damage to surrounding tissues [4]. Following the acute phase, the stroke progresses into a chronic phase, which is marked by cellular damage and secondary complications. This phase hinders the brain’s ability to undergo cellular plasticity and regeneration, which is essential for recovery. The extent of the injury and the brain’s adaptive responses during this chronic phase are influenced by many factors, including the initial severity of the stroke, the region of the brain affected, and the presence of underlying health conditions [5]. Understanding the dynamics of these phases is crucial for developing effective therapeutic interventions aimed at enhancing neuronal survival and promoting recovery [6].

Understanding of the mechanisms of stroke pathology and recovery is crucial for developing effective biomarkers for diagnosis or prognosis and for identifying novel therapeutic targets. This is influenced by understanding the mechanisms of stroke pathology and recovery is crucial for developing effective biomarkers for diagnosis or prognosis and for identifying novel therapeutic targets [7]. This is influenced by a complex interplay of genetic, environmental, and cellular factors, with recent studies highlighting the critical role of non-coding RNAs (ncRNAs), particularly microRNAs (miRNAs), in modulating these processes.

miRNAs are short, non-coding transcripts of 19-25 nucleotides in length [8,9,10,11]. By directly binding to messenger RNAs and other ncRNAs, they regulate the function of a wide range of genes through RNA degradation or inhibition of translational processes. An essential mechanistic feature of these transcripts is their partial complementarity to their target genes [8,12,13,14]; thus, a single miRNA can target multiple RNA sequences, and several miRNAs can regulate the same mRNA transcript [14,15,16,17,18,19]. miRNAs play crucial roles in various biological processes by binding to different target genes, influencing cell differentiation, proliferation, apoptosis, cell cycle progression, invasion, metastasis, and immune responses [20], contributing to the development and progression of diseases [21,22]. Accordingly, it can be assumed that miRNAs should have significant promises as biomarkers and therapeutic targets in stroke management [8,17]. Their high stability in tissue or in various biological fluids, and their ability to reflect pathological changes make them valuable tools for improving diagnostic accuracy, prognostic assessments, and therapeutic strategies [23,24].

A growing number of studies are concentrating on evaluating miRNA profiles in stroke or restoring their normal expression levels (miRNA mimics or antagomirs) for their deeper mechanistic role or as a therapeutic approach.

This review summarizes recent research on the shared and distinct functions of the miR-181 family members in the context of stroke. We emphasize the roles in stroke pathogenesis and biomarker discovery and support the development of innovative miRNA-based therapeutic approaches. miR-181 plays central roles in the regulation of neuroinflammation, apoptosis, and neuronal repair, making them potential candidates for novel neuroprotective and reparative strategies. Understanding and harnessing these small non-coding RNAs could pave the way for breakthroughs in stroke therapy, addressing both the immediate and long-term challenges associated with this devastating condition.

**miR-181 family members.** MiR-181 family plays a significant role in fine-tuning gene expression in response to physiological and pathological stimuli [25,26]. Alterations in the expression levels of these miRNAs have been linked to various diseases, including cancers, neurodegenerative disorders, and cardiovascular diseases [25,26,27,28,29].

**Figure 1:**
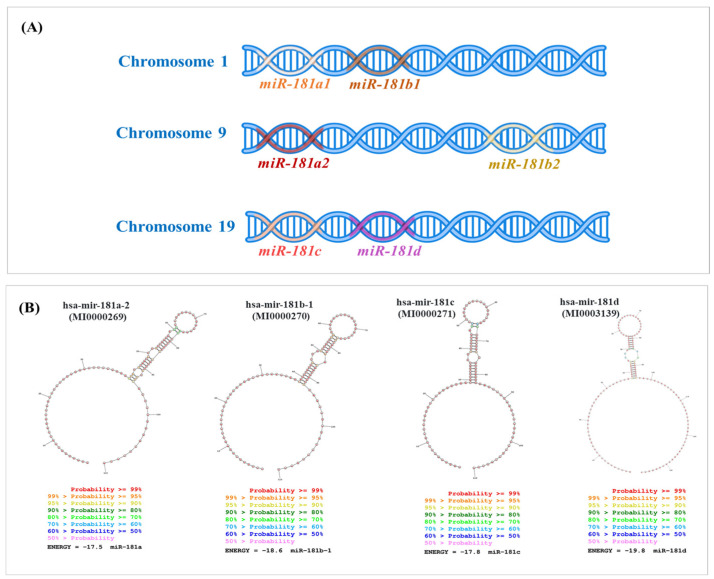
**Genomic distribution and the secondary structures of the miR-181 family members across human chromosomes.** (**A**) Location of miR-181 family members across three different human chromosomes. Chromosome 1 harbors the miR-181a1 and miR-181b1 transcript, depicted as adjacent segments on the DNA strand. Chromosome 9 contains the miR-181a2 and miR-181b2 transcripts, which are similar to those on chromosome 1. Chromosome 19 includes the miR-181c and miR-181d, positioned next to each other on the DNA strand. (**B**) Sequences for mature hsa-miR-181 family members were downloaded from miRBase (http://mirbase.org, 27 September 2024) and used as input to generate the secondary structures by RNA structure software [36].

miR-181a and miR-181b, located on chromosomes 1 and 9, can originate from two distinct precursor molecules, contributing to their diversity in biogenesis and functional roles. Meanwhile, miR-181c and miR-181d are clustered on chromosome 19 [30,31] (Figure 1). Despite sharing the same seed sequence, each family member can selectively bind to distinct target genes, influencing various cellular functions, including apoptosis, proliferation, and immune responses [25,32]. Their capacity to modulate critical signaling pathways highlights their potential as biomarkers and therapeutic targets [32].

Due to its diverse target genes, miR-181s control multiple cellular pathways crucial for stroke pathogenesis, including those involved in neuronal development, apoptosis, inflammation, and more [25]. Each member of the miR-181 family—miR-181a, miR-181b, miR-181c, and miR-181d—participates in nuanced interactions with distinct sets of target genes, thereby influencing complex cellular networks critical for understanding and potentially treating stroke [25]. This versatility in target gene binding highlights the significance of miR-181s as key regulators in maintaining cellular homeostasis and responding to the challenges presented by stroke [33,34]. Their role in these processes underscores their potential as valuable targets for therapeutic interventions aimed at mitigating the impact of stroke and promoting recovery, considering that it is one of the transcripts highly expressed in brain tissue [35].

**miR-181s single nucleotide polymorphisms (SNPs)-implications in stroke.** The miR-181 family, through various polymorphisms (SNP), was demonstrated to be related to stroke risk [33,37], exemplified in Table 1. The miR-181a expression levels in the peripheral blood mononuclear cells were found to be significantly higher in IS patients than in the controls. Among IS patients, those with the rs322931 GA + AA genotype had significantly higher miR-181a expression levels compared to those with the rs322931 AA genotype, while no significant difference was observed in miR-181a expression among controls with rs322931 genotypes [37]. In a study involving 509 IS patients and 668 healthy controls, genotyping revealed that individuals with the miR-181b rs322931 CT and CT/TT genotypes had a higher risk of IS compared to those with the CC genotype [33]. The rs322931 CT/TT genotypes were also associated with higher levels of low-density lipoprotein cholesterol (LDL-C), a known risk factor for IS. Logistic regression analysis confirmed that these genotypes are significant risk factors for IS, alongside other factors such as hypertension and total cholesterol levels. Additionally, a dual-luciferase reporter assay demonstrated that the rs322931 T allele results in lower luciferase activity than the C allele, suggesting a functional impact of this SNP on miR-181b expression and activity. These findings highlight the potential role of miR-181b rs322931 in contributing to the susceptibility to ischemic stroke [33]. A study in the Chinese population found that the miR-181c rs8108402 SNP is significantly associated with IS risk, with the CT genotype increasing and the TT genotype decreasing the risk compared to the CC genotype. Additionally, the miR-181c rs8108402 polymorphism correlates with low-density lipoprotein cholesterol (LDL-C) levels in IS patients, and significant differences in the expression of miR-181a, miR-181b, and miR-181c between IS patients and controls suggest that miR-181 gene clusters could serve as predictive and therapeutic targets for IS [38]. Therefore, investigating how miR-181 SNPs interact with other genetic variants and environmental factors (e.g., diet, lifestyle, and comorbid conditions) to modulate IS risk could provide insights into the complex interplay between genetics and environment in stroke susceptibility.

**Alteration of miR-181s expression levels in stroke.** In the context of stroke, dysregulation of miR-181 expression was observed in both acute and chronic phases of the disease [7,35,39]. Importantly, these alterations of the expression levels in blood or plasma emphasize the role of biomarkers in the early diagnosis and prognosis of stroke [34,35,39,40]. Studies have reported that miR-181a and miR-181b levels are often upregulated in plasma as a response to ischemic conditions, potentially reflecting a protective mechanism that aims to mitigate neuronal damage [26,41]. miR-181a-5p is highly expressed in the serum of acute cerebral infarction (ACI) patients, in middle cerebral artery occlusion (MCAO) mice, and in N2a cells in oxygen-glucose deprivation/reperfusion (OGD/R) [40].

A profiling study of 754 miRNAs analyzed plasma samples collected at 5, 15, and 30 days post-ischemic stroke [35]. The study focused on identifying key miRNA biomarkers in stroke diagnosis and prognosis. miR-181a was part of the specific signature, being identified as altered at all time points evaluated [35]. For validation, plasma samples from 48 stroke patients (15 days post-event) were compared with samples from 55 healthy controls, being validated the expression level for miR-181a [35].

A lymphocyte miRNA microarray analysis of IS patients identified members of the miR-181 family, including hsa-miR-181a/c/d, among the top 44 differentially expressed miRNA [39]. MiR-181c exhibited the most significant change among its family members [39]. An additional validation step was done, RT-PCR analysis confirmed that miR-181c levels were decreased in the plasma of stroke patients compared to healthy controls, indicating the clinical relevance of this transcript in stroke [39].

In an animal study, miR-181b-5p was selectively upregulated in the plasma of rats subjected to collagenase-induced hemorrhagic stroke [7]. This finding highlights the potential of miR-181b-5p as a biomarker for distinguishing hemorrhagic stroke from ischemic stroke, emphasizing the importance of precise time point measurement to enhance the reliability of miRNAs as stroke biomarkers [7]. Distinct miRNA expression patterns in response to different types of stroke were observed. MiR-181b-5p, along with miR-150-5p, let-7b-5p, and let-7c-5p, showed significant upregulation in the plasma of rats following collagenase-induced hemorrhagic stroke [7]. In contrast, other miRNAs, including miR-16-5p, miR-101a-3p, miR-218-5p, and miR-27b-3p, were specifically upregulated three hours after permanent middle cerebral artery occlusion (MCAO) [7]. In another animal study, the expression levels of miR-181a in the ischemic core increased while they decreased in the penumbra at different durations of reperfusion following 1 h of middle cerebral artery occlusion (MCAO) in mice [42]. Similarly, miR-181b, miR-181c, and miR-181d exhibit comparable changes, with increased expression in the ischemic core and decreased levels in the penumbra after ischemia [42]. In Table 2 are presented a summary of studies investigating miR-181s expression level in IS.

**miR-181s in correlation with oxidative stress.** Oxidative stress plays a critical role in causing cellular and molecular damage during post-ischemic insult and has therapeutic potential for preventing secondary brain damage after stroke [6]. Oxidative stress arises from the imbalance between oxidation and antioxidation during injury processes and self-regulation mechanisms, primarily driven by reactive oxygen species (ROS) production. During acute stroke, the excessive production of ROS can trigger early inflammation, leading to the activation of immune cells and ultimately resulting in cell death post-stroke.

**Figure 2:**
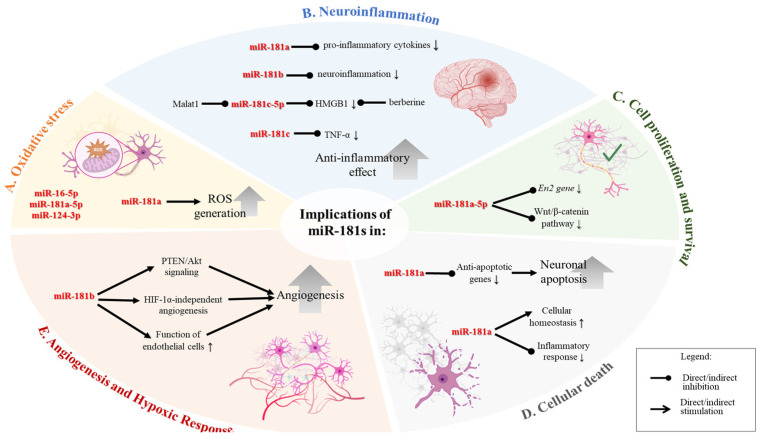
**Biological roles and implications of miR-181 family members in cellular processes**, illustrating the diverse biological functions and implications of the miR-181 family across various cellular processes, particularly in the context of oxidative stress, neuroinflammation, cell proliferation and survival, cellular death, and angiogenesis. (**A**). **Oxidative Stress.** Upregulation of miR-181a leads to increased production of reactive oxygen species (ROS), contributing to oxidative stress within cells. (**B**). **Neuroinflammation.** miR-181a downregulates the expression of pro-inflammatory cytokines, reducing neuroinflammation. miR-181b acts to decrease neuroinflammation. miR-181c reduces levels of TNF-α, a key inflammatory cytokine. miR-181c targets HMGB1 and is regulated by Malat1, contributing to an anti-inflammatory effect. (**C**). **Cell Proliferation and Survival.** miR-181a inhibits the En2 gene and the Wnt/β-catenin pathway. Therefore, the downregulation of the miR can regulate cell proliferation and survival. (**D**). **Cellular Death.** miR-181a/d downregulates anti-apoptotic genes, leading to increased neuronal apoptosis. It also plays a role in maintaining cellular homeostasis and reducing inflammatory responses, which may influence cell survival. (**E**). **Angiogenesis and Hypoxic Response**. miR-181b modulates angiogenesis through PTEN/Akt signaling and HIF-1α-independent pathways, enhancing the function of endothelial cells and promoting the formation of new blood vessels. Figure created in BioRender.com.

Astrocytes transfected with pri-miR-181ab showed a significant rise in ROS generation, though mitochondrial membrane potential remained unaffected in unstressed cells for up to 48 h-post transfection [42]. These findings suggest that miR-181a contributes to cell death under ischemic conditions by increasing ROS production, thereby worsening the injury [42], Figure 2A. The suppression of miR-181d reduced apoptosis and oxidative stress in neuroblastoma cells treated with OGD/R, whereas the overexpression of miR-181d increased both [43]. miR-181a may also be linked to elevated levels of chaperones, especially GRP78, a typical adaptation to chronic stress in tumor cells that suppresses apoptosis, impacting cerebral ischemia outcomes [42].

A bioinformatic study revealed mir-16-5p, hsa-mir-181a-5p, and hsa-mir-124-3p as key miRNAs, and the main target genes to be related to oxidative stress and inflammatory reaction [44].

**miR-181 in neuroinflammation and immune response regulation.** Neuroinflammation is a critical driver of brain damage after a stroke [34]. The miR-181 family has an important role in regulating the expression of inflammatory mediators and immune cell activation, thereby affecting overall stroke outcomes [45,46,47,48].

A study done by Xie et al. reported that miR-181a has a significant role in promoting cellular survival in vitro by repressing inflammatory processes in macrophages and monocytes [49]. Their study demonstrated that miR-181a downregulates key inflammatory pathways, leading to reduced production of pro-inflammatory cytokines and mediators. By targeting specific signalling molecules and transcription factors involved in the inflammatory response, miR-181a effectively decreases the inflammatory environment, thereby protecting cells from inflammation-induced damage. This anti-inflammatory action of miR-181a enhances the survival of macrophages and monocytes [49]. It suggests potential therapeutic applications in conditions where inflammation is detrimental, such as IS.

The MALAT1/miR-181c-5p/HMGB1 axis has been identified as a novel key pathway in stroke inflammation. The miR-181c-5p expression was negatively regulated by MALAT1. The miR-181c-5p and HMGB1 levels were inversely correlated through transfection experiments. MALAT1/miR-181c-5p/HMGB1 axis may be a key pathway of antiinflammation induced by berberine, highlighting its potential as a therapeutic targets for modulating inflammation and improving stroke outcomes [48]. MALAT1 was proven to act as a competing endogenous RNA (ceRNA) to facilitate the berberine-mediated inhibition of HMGB1 by sponging miR-181c-5p in post-stroke inflammation [48].

Transfection of neuronal cells with miR-181c has been shown to inhibit the expression of TNF-α, a key pro-inflammatory cytokine, during the acute phase of ischemic injury [50]. This inhibition alleviates microglial activation and neuronal cell death, particularly apoptosis. This highlights the therapeutic potential of miR-181c in modulating inflammation and protecting neurons during ischemic injury [50]. miR-181c-5p suppresses stress-induced inflammatory responses, which are notably downregulated following a stroke. This downregulation could contribute to the exacerbation of neuroinflammation and subsequent neuronal damage post-stroke [34].

In intracerebral hemorrhage (ICH), post-ICH inflammation significantly affects clinical outcomes. An integrated miRNA-seq and mRNA-seq study followed by a complex bioinformatic analysis in a swine model identified miR-181a as a key anti-inflammatory regulator. It showed decreased levels after ICH and a strong connection with IL-8 and monocytes, suggesting its potential as a therapeutic target for managing post-ICH inflammation [51]. Refer to Figure 2B for a schematic representation of the mechanisms underlying representative examples of how miR-181 family members are implicated in neuroinflammation.

**Cell proliferation and survival.** In the context of stroke, members of the miR-181 family are critically involved in regulating cell proliferation and survival [37,42,52]. These transcripts modulate cellular responses to injury by influencing key signalling pathways that govern cell fate decisions. Their role in promoting cell survival and mitigating apoptosis can be particularly beneficial in the aftermath of a stroke, where neuronal loss and tissue damage are prevalent. By affecting these pathways, miR-181 family members have the potential to enhance neuronal survival, support recovery, and improve overall outcomes in stroke patients [37].

Increased miR-181a levels exacerbate ischemia-like injury by significantly increasing glucose deprivation [GD] induced cell death in primary astrocyte cultures. This was demonstrated by transfecting cells with a pri-miR-181ab construct, which resulted in a doubling of miR-181a levels and a marked increase in cell death compared to cells transfected with a seed mutant construct [42].

Down-regulation of miR-181a-5p helps prevent cerebral ischemic injury by upregulating the gene *En2* and activating the Wnt/β-catenin pathway under OGD/R (oxygen-glucose-deprivation/reoxygenation) conditions (Figure 2C). This process enhances neuroprotection by promoting cell survival and mitigating damage during ischemic events. The activation of the Wnt/β-catenin pathway is particularly significant as it is involved in various cell survival and neurogenesis mechanisms, highlighting the therapeutic potential of targeting miR-181a-5p in ischemic stroke treatment [40]. The miR-181a signaling is involved in mitochondrial function recovery, particularly in ischemic-hypoxic preconditioned stem cells, which improves neuronal resilience and function post-stroke [52].

**Apoptosis, necroptosis, and pyroptosis in stroke.** Apoptosis, necroptosis, and pyroptosis are distinct forms of programmed cell death, each regulated by different mechanisms. Apoptosis is controlled by caspase-3. Necroptosis involves receptor-interacting protein kinases and is mediated by receptor-interacting protein kinases. Pyroptosis is driven by caspase-1 and is associated with inflammation. A recent bioinformatic study identified key nodes in apoptosis, necroptosis, and pyroptosis, with miR-181 family members as central elements in the ceRNA regulatory network [41].

Moon et al. have shown that silencing miR-181a attenuated neuronal apoptosis induced by forebrain ischemia. This study indicated that reducing miR-181a levels in neurons can protect against ischemia-induced cell death, suggesting that miR-181a plays a pro-apoptotic role in the context of forebrain ischemia [53].

The miR-181a was linked to apoptosis during the first month after stroke, while other miRNAs indicated a shift toward synapse regulation and neuronal protection by day 30. These findings imply that reduced cellular proliferation may persist for at least 30 days after stroke, highlighting specific miR-181a as a key regulator that could play a role in neural repair in humans [35]. Also, miR-181a targets and downregulates anti-apoptotic genes, potentially influencing cell death pathways activated during ischemic stroke. Understanding these regulatory mechanisms could offer insights into how to protect neurons from ischemia-induced apoptosis.

Research involving ischemic-hypoxic preconditioned olfactory mucosa-derived mesenchymal stem cells (IhOM-MSCs) has demonstrated that miR-181a upregulation is associated with protective effects against ischemic stroke. Transplantation of ischemic-hypoxic preconditioned IhOM-MSCs has been shown to attenuate both apoptotic and pyroptotic cell death following ischemic stroke. This therapeutic approach capitalizes on the ability of IhOM-MSCs to mitigate cell death mechanisms through various pathways. Specifically, IhOM-MSCs have been observed to modulate the expression of key regulatory molecules such as miR-181a, which enhances downstream targets involved in maintaining cellular homeostasis and suppressing inflammatory responses [52].

Pyroptosis shares similarities with apoptosis but differs in its mechanisms, relying on the inflammation-related protein caspase-1 rather than the apoptosis-related caspase-3. Ischemic-hypoxic preconditioning enhances mitochondrial function recovery in transplanted olfactory mucosa mesenchymal stem cells via miR-181a signaling in ischemic stroke [52]. Figure 2D provides a schematic depiction of representative mechanisms highlighting the involvement of miR-181 family members in cellular death.

**Angiogenesis and hypoxic response in stroke.** Angiogenesis, forming new blood vessels, is crucial for tissue repair and recovery following ischemic events. miR-181b’s role in promoting angiogenesis could potentially aid in restoring blood supply to ischemic brain regions, thereby mitigating stroke-induced damage, and promoting recovery. miR-181b has been shown to enhance angiogenesis via hypoxia in a HIF-1α-independent manner. Hypoxia and ischemia trigger microglial activation and release pro-inflammatory cytokines and neurotoxic factors. This response is part of the body’s attempt to deal with the damage caused by reduced oxygen supply, but it can lead to further neuronal injury and inflammation. The activation of microglia and subsequent release of soluble mediators [50].

A recent study evaluated the effects of miR-181b on brain microvascular endothelial cells (BMECs) under oxygen-glucose deprivation (OGD), an in vitro model mimicking ischemic stroke conditions. In this condition, the overexpression of miR-181b in BMECs subjected to OGD significantly restored cell proliferation and enhanced angiogenesis. These findings suggest that miR-181b may play a crucial role in vascular repair and regeneration following ischemic stroke via PTEN/Akt signaling pathway [46]; this being validated on rat models of focal cerebral ischemia, the overexpression of miR-181b reduced infarction volume, promoted angiogenesis in the ischemic penumbra, and improved neurological function [46].

Ischemic-hypoxic preconditioning has been shown to enhance the recovery of mitochondrial function in transplanted olfactory mucosa mesenchymal stem cells via miR-181a signaling. This preconditioning promotes the resilience and functional integration of the transplanted stem cells, leading to better outcomes in terms of mitochondrial function and potentially overall cell survival and repair in the ischemic brain environment [52]. Exosome-derived miR-181b has been shown to promote the angiogenesis of brain microvascular endothelial cells [BMVECs] after hypoxia in vitro. Endothelial cells are crucial for maintaining the blood-brain barrier and supporting neuronal health. Enhancing the function and repair of these cells through miR-181b could be a therapeutic strategy to support brain recovery post-stroke [46,54]. Exosomal microRNA-181b-5p (181b-Exos) derived from adipose-derived stem cells (ADSCs) plays a pivotal role in regulating post-stroke angiogenesis. The angiogenic potential of brain microvascular endothelial cells (BMECs) was done by targeting and downregulating transient receptor potential melastatin 7 [TRPM7], leading to increased expression of HIF1α and VEGF [54]. Figure 2E summarizes the mechanisms by which miR-181b regulates angiogenesis and hypoxic processes.

**Therapeutic potential of miR-181.** The miR-181 family members were proved to be valuable targets for therapeutic interventions in stroke [34,42,55]. Modulating the levels of specific miRNAs in the miR-181 family could help develop treatments to reduce stroke severity, enhance recovery, and prevent recurrent strokes, considering their complex biological role and implications in cellular response (Figure 2).

Post-stroke treatment with miR-181 antagomir represents a promising therapeutic strategy for mitigating injury and enhancing recovery following focal cerebral ischemia. The miR-181 has been implicated in promoting neuronal apoptosis and pyroptosis, contributing to the extent of brain damage after ischemic events. By inhibiting miR-181 through an antagomir, this treatment reduces these detrimental processes. Experimental studies in mice have shown that miR-181 antagomir reduces the extent of brain injury and improves long-term behavioral outcomes. This suggests that targeting miR-181 can effectively protect neurons and support neurological recovery, making miR-181 antagomir a potential therapeutic approach for improving post-stroke recovery [56].

Research indicates that miR-181 family members play a crucial role in mediating neuronal injury caused by oxygen-glucose deprivation and reoxygenation (OGR/R). Experiments conducted in vitro on N2A cells demonstrated that the downregulation of miR-181b alleviates injury caused by OGD. Reduced miR-181b expression led to decreased cell death and improved cell survival, indicating its role in neuronal protection under ischemic conditions [45]. OGD study model in BV-2 cells results in the upregulation of tumor necrosis factor-α [TNF-α] and the downregulation of miR-181c. This suggests that OGD, a widely used model for ischemic conditions, promotes inflammatory responses through increased TNF-α expression while reducing miR-181c levels, which normally act to suppress such inflammation [50]. miR-181d directly targets DOCK4, a gene involved in cell signaling and cytoskeletal organization. The suppression of DOCK4 by miR-181d further contributes to neuronal injury under OGD/reoxygenation conditions [43].

Inhibition of miR-181a has been found to promote neurogenesis in neural stem cells. This suggests that miR-181a negatively regulates the proliferation and differentiation of neural stem cells and its inhibition can enhance the generation of new neurons [57]. Understanding these interactions provides valuable insights into the molecular mechanisms underlying neuronal injury and highlights miR-181a and miR-181d as potential targets for therapeutic interventions aimed at reducing brain damage following ischemic events [37,40,45]. Another study revealed neuroprotective mechanisms mediated through the upregulation of En2, a gene involved in neural development. Additionally, the suppression of miR-181a-5p activates the Wnt/β-catenin pathway, a critical signaling pathway that promotes cell survival, proliferation, and differentiation. By upregulating En2 and activating the Wnt/β-catenin pathway, the downregulation of miR-181a-5p helps mitigate the damage caused by cerebral ischemia, highlighting its potential as a therapeutic target for stroke treatment [40].

The miR-181b is implicated in various aspects of neural function, including neuroprotection, synaptic plasticity, and neurogenesis. The miR-181b has been shown to target and downregulate PirB [immunoglobulin-like receptor B] expression, which regulates axon growth and plasticity in the nervous system [58]. It was observed that the upregulation of miR-181b as the effect of electroacupuncture therapy may suppress PirB expression, thereby promoting axon regeneration synaptic plasticity and ultimately improving functional recovery in stroke-affected areas of the brain [58]. The interaction between miR-181b and PirB represents a promising avenue for therapeutic intervention in stroke rehabilitation.

In a mouse model study, supplementation with a miR-181c-5p mimic after stroke can restore its expression levels in the context of post-stroke social isolation. This restoration enhances the interactions of RNA-induced silencing complexes (RISCs) with their target genes, leading to a reduction in inflammatory gene expression and potentially improving recovery outcomes [34].

Transplantation of IhOM-MSCs has shown promise in mitigating these effects by upregulating protective genes like GRP78 and Bcl-2 via miR-181a, suggesting that IhOM-MSCs could be an effective future therapy for ischemic stroke [52]. Therefore, this represents a promising strategy for reducing neuronal loss and improving outcomes in ischemic stroke, highlighting their potential as a novel therapeutic intervention in stroke treatment protocols. Please refer to Table 3 for a detailed summary of studies investigating the mechanistic roles of miR-181 family members in various in vitro and in vivo stroke models, including their therapeutic applications and observed outcomes.

The therapeutic potential of the miR-181 family in stroke will be elucidated as additional evidence emerges, such as preclinical studies demonstrating its role in modulating neuroinflammation, oxidative stress, apoptosis, and angiogenesis. Significant challenges still remain in translating these findings into clinical applications. The most significant translational issue is the efficient delivery of miRNA-based therapies across the blood-brain barrier (BBB). Delivery systems such as nanoparticles, exosomes, and direct intracerebral injections show potential as proofs of concept, yet their scalability, safety, and efficacy in humans are not validated. Additionally, miRNAs like the miR-181 family have broad target profiles, raising concerns about off-target effects and unintended modulation of non-stroke-related pathways, which could result in adverse safety outcomes. The heterogeneity of stroke pathology, influenced by factors such as stroke subtype, age, gender, and comorbidities, further complicates the development of a universal therapeutic approach. Personalized medicine strategies that consider these variables will be necessary.

Future research should address these problems by focusing on advanced delivery mechanisms that can cross the BBB with high specificity and efficiency. Additionally, there is a need for long-term preclinical studies to evaluate the safety, efficacy, and sustained impact of miR-181 modulation in diverse models of stroke. Combining miR-181-targeted therapies with existing interventions, such as thrombolytics or neuroprotective agents, may enhance therapeutic outcomes. Early-phase clinical trials are needed to assess the feasibility of miR-181-based interventions in human populations, focusing on stratified patient groups and robust biomarker-guided monitoring.

## 2. Conclusions

The miR-181 family represents a significant area of interest in stroke research due to its involvement in key processes such as angiogenesis, inflammation, cell survival, and endothelial function. The versatility in target gene binding highlights the significance of miR-181s as key regulators in maintaining cellular homeostasis and responding to the challenges presented by stroke. Their role in these processes underscores their potential as valuable targets for therapeutic interventions to improve stroke patients’ prognosis and functional recovery.

Our review has largely concentrated on the role of miR-181 family members in ischemic stroke, but also their potential involvement in hemorrhagic stroke. As highlighted earlier, distinct expression patterns of miR-181 members, particularly miR-181b-5p, have been observed in models of hemorrhagic stroke, suggesting potentially different roles in the pathogenesis of this stroke subtype compared to ischemic stroke [7]. While the precise mechanisms remain to be fully elucidated, the existing data, including alterations in inflammatory pathways, imply that miR-181 family members may contribute to the unique pathophysiology of hemorrhagic stroke. Further research examining miR-181 in hemorrhagic stroke models is needed to clarify these distinctions and explore the full therapeutic potential of targeting this miRNA family across all stroke subtypes. Also, as mentioned before, miR-181a may have a role as an anti-inflammatory regulator, with decreased levels after ICH [51].

Understanding the full therapeutic potential of these transcripts in stroke requires further investigation. Targeting miR-181a, in particular, holds significant promise for enhancing stroke outcomes and neurological recovery. Developing pharmacological strategies to either modulate miR-181a expression or alter its downstream effects is a critical next step. While this approach shows considerable potential for ischemic stroke treatment, extensive research, including clinical trials, is necessary to validate its clinical utility.

An emerging area of inquiry is the potential influence of age and gender on miR-181’s activity. Preliminary evidence indicates that miR-181 regulation may vary between males and females, as well as across the lifespan, likely due to hormonal and age-related influences. However, our understanding of miR-181’s precise function in these contexts is still in its infancy. Looking at the age- and gender-specific expression patterns of miR-181, as well as differences between ischemic and hemorrhagic strokes, could offer insights into its regulatory mechanisms and potential for tailored stroke therapies, opening the field to personalized treatments.

## Figures and Tables

**Table 1:** Short description of the frequent SNP and correlation with clinical parameters in ischemic stroke (IS).

SNP Locus	Polymorphism	Relevant Findings of the Study and Correlation with Expression Level or Other Relevant Clinical Data	Reference
miR-181a	rs322931	Variant related to a higher risk of IS	[37]
miR-181b	rs322931	MEG3/miR-181b polymorphisms for risk stratification	[33]
miR-181c	rs16927589, rs77418916, rs8108402	Association between the polymorphism and expression level of the family miR-181s	[38]

**Table 2:** Summary of miR-181s expression levels in different biological specimens of IS.

Transcript Member	Biological Specimens	Evaluation Methos	Findings	Ref.
↑ miR-181a-3p ↑ miR-181a-5p	IS samples at 48h, 5, 15, and 30 days post-IS (*n* = 55) versus healthy control plasma	Profiling study (miRNA microarray) Validation cohort qRT-PCR: IS at 48h respectively, 15-days post-IS (*n* = 55) to healthy control samples (*n* = 48)	↑ miR-181a part of a biomarker signature; involved in apoptosis, synapse regulation and neuronal protection.	[35]
↑ miR-181a-5p	IS (*n* = 81) and healthy controls (*n* = 42) plasma samples; MCAO models; OGD/R-induced N2a cells MACAO model versus sham group; OGD/R-induced N2a cells versus control N2a cells	qRT-PCR	correlated to the pathological of IS	[40]
↓ miR-181a-5p ↓ miR-181a-3p ↓ miR-181c-5p ↓ miR-181c-3p ↓ miR-181d-5p	IS versus healthy control lymphocytes	Profiling study (miRNA microarray)	part of altered miRNA signature specific for IS	[39]
↓ miR-181c-5p ↓ miR-181d-5p	IS (*n* = 10) and healthy controls (*n* = 7) plasma	qRT-PCR	miR-181c correlated with clinical parameters of IS (lymphocyte percentage, neutrophil number)	[39]

IS: ischemic stroke. qRT-PCR: quantitative real-time polymerase chain reaction. MACO: middle cerebral artery occlusion. OGD/R: oxygen-glucose-deprivation/reoxygenation.

**Table 3:** Studies investigating the role of miR-181 family members as therapeutic target in various in vitro and in vivo stroke models.

In Vitro and/or In Vivo Methods	Therapeutic Approaches/Delivery System	Fold Change	Observation Related to Therapeutic Role of Modulation of This Transcript	Ref.
Astrocyte				
N2a cells Astrocytes, Male Sprague–Dawley rats	10 pmol of miR-181a mimic and 20 pmol of inhibitor; cationic lipid DOTAP 1:3 infused stereotactically just outside the left hippocampus (from bregma −3.8 mm, M-L 2.0 mm, deep 2.5 mm) at 1 μL/minute, maximal total volume 16 μL via a burr hole	↑ miR-181a	miR-181a is upregulated in the infarct core and downregulated in the penumbra after focal ischemia. Inhibition of miR-181 reduces forebrain ischemia-induced neuronal loss and activated cell death mechansims by caspases 3 and 7 activation	[53]
Mouse primary astrocyte cultures C57BL/6J mice or TNFR1/TNFR2 double knockout (DKO)	miR-181c and miR-181b hairpin inhibitors/Lipofectamine	↑ miR-181b/c/d	regulate cell proliferation and neuroinflammation; affect cytokine production cell death and inflammation responses genes; direct targets MeCP2 and XIAP	[45]
Neurons				
N2a cells, primary mouse neuronal culture	miR-181a mimics and inhibitors Lipofectamine 2000	N/A	Inhibition of miR-181a reduces forebrain ischemia-induced neuronal loss, affects apoptosis via BCL2 and GLT-1	[59]
Primary hippocampal microglial and hippocampal neuronal rat cells	miR-181a mimics and inhibitors Lipofectamine 2000	N/A	microglia-mediated neuronal apoptosis by targeting TNF-α	[50]
Microglia				
BV-2 microglial cell line and primary cultured rat microglial cells	miR-181c mimics/inhibitors and negative control/Lipofectamine 2000		miR-181c regulates TLR4 expression; miR-181c inhibits NF-κB activation and the downstream production of proinflammatory mediators	[47]
BV-2 microglial cells and HEK-293T cells	Lenti-miR-181a-5p inhibitor, Lenti-NC, sham, miR-181a and NC inhibitor Lipofectamine 2000		rescues HMGB1 inhibition induced by Malat1 downregulation	[48]
BV2 microglial cells and Neuro-2a cells	Lipopolysaccharide (LPS)/hydrogen peroxide (H2O2); miR-181c mimic/inhibitor (50 nM/L), Lipofectamine RNAiMAX	↓ miR-181a/c/d	MiR-181c agomir inhibits proliferation and induces apoptosis of BV2 microglial cells upon oxidative stress and inflammation, accelerates apoptosis of neuronal cells co-cultured with microglial cells	[39]
Cerebral ischemia				
OGD/R N2a cell culture model and MCAO in vivo model	miR-181a inhibitor si NC, Lipofectamine 2000	↑ miR-181a	Down-regulation of miR-181a-5p prevents cerebral ischemic injury by upregulating En2 and activating the Wnt/β-catenin pathway	[40]
OGD/R N2a cell culture model and MCAO in vivo model	miR-181b antagomir and negative control/intracerebroventricular infusion (5 pmol/ll, at a rate of 1 ll/hr)	↓ miR-181b in Response to Ischemic Exposure	Downregulation of miR-181b Reduces OGD Induced N2A Cell Injury; miR-181b Regulates HSPA5 and UCHL1 Protein Levels Through Binding to 30 -UTR mRNAs region	[45]
Neuro2A and HEK293T cells; MCAO in vivo model	AMO181a-chol loaded onto RBP-Exo		neuroprotective effects in the ischemic brain	[59]
OGD/R N2a cell culture model; MCAO in vivo model	siRNA SNHG6, mR-181c mimic/inhibitor and negative control, Lipofectamine 2000	N/A	SNHG6 directly binds to miR-181c-5p and negatively regulates its expression; miR-181c-5p targets the 3′UTR of BIM and negatively regulates the expression of BIM; ceRNA- SNHG6-miR-181c-BIM and promote cell apoptosis	[60]
SH-SY5Y cell in OGD/R condition and MCAO in vivo model	miR-181a inhibitor Lipofectamine 2000	↑miR-181a in the MCAO model versus sham	PTEN overexpression reduced cell apoptosis and oxidative stress induced by miR-181a upregulation under an OGD/R condition	[37]
OGD/R N2a cell culture model; Transient MCAO model in rats	miR-181a inhibitor si NC, Lipofectamine 2000	↑miR-181d	miR-181d regulates cerebral ischemia/reperfusion injury by negatively targeting DOCK4 under OGD/R	[43]
BMVECs and MCAO in vivo model	miR-181b-5p mimic si inhibitor	-	regulate angiogenesis and neurological function via PTEN/Akt; miR-181b-5p agomir promotes neurological function recovery and reduces infarct volume in MCAO rats	[46]
MCAO or intracerebroventricular (ICV)	Lenti-miR-181c-5p, miR-181c-5p inhibitor Lenti-NC or NC		Malat1/miR-181c-5p/HMGB1 axis a key pathway in stroke	[48]
Hemorrhagic stroke				
Plasma MACAO-collagenase-induced hemorrhagic stroke	miR-181a-5p	↑ miR-181a	time-dependent upregulation of miR-150-5p and miR-181b-5p in the plasma	[7]
Ischemic-hypoxic preconditioned olfactory mucosa-derived mesenchymal stem cells (IhOM-MSCs) and MSCs and SH-SY5Y neurons in a co-culture system and MCAO in vivo model	miR-181a mimic and inhibitor	N/A	IhOM-MSCs mediated the upregulation of the downstream target genes GRP78 and Bcl-2 by miR-181a to protect mitochondrial function and inhibit apoptosis and pyroptosis of neurons in the ischemia/reperfusion injury model.	[52]
Mice Intracerebroventricular Infusion (ICV) and Intravenous Injection (IV) of 181a Antagomir	miRNA-181a antagomir (3 pmol/gram in 2 μL final volume) and a negative control: mismatched (MM)-for miR-181a antagomir mixed with the cationic lipid DOTAP (4 μL)	↑ miR-181a in stroke	Post-stroke treatment with miR-181a antagomir reduces infarction size, reduces long-term neurobehavioral deficits and inflammation; targets BCL2 and XIAP; both administration routes are effective.	[56]

NC: negative control. OGD/R: oxygen-glucose-deprivation/reoxygenation. MCAO: Mice middle cerebral artery occlusion in vitro model. ICV: Mice Intracerebroventricular Infusion. IV: Intravenous Injection.

## References

[1] Benjamin E.J., Muntner P., Alonso A., Bittencourt M.S., Callaway C.W., Carson A.P., Chamberlain A.M., Chang A.R., Cheng S., Das S.R. (2019). Heart Disease and Stroke Statistics-2019 Update: A Report From the American Heart Association. Circulation.

[2] Feigin V.L., Roth G.A., Naghavi M., Parmar P., Krishnamurthi R., Chugh S., Mensah G.A., Norrving B., Shiue I., Ng M. (2016). Global burden of stroke and risk factors in 188 countries, during 1990-2013: A systematic analysis for the Global Burden of Disease Study 2013. Lancet Neurol..

[3] Grefkes C., Fink G.R. (2020). Recovery from stroke: Current concepts and future perspectives. Neurol. Res. Pract..

[4] Turner D.A., Yang W. (2021). Phase-specific manipulation of neuronal activity: A promising stroke therapy approach. Neural Regen. Res..

[5] Salaudeen M.A., Bello N., Danraka R.N., Ammani M.L. (2024). Understanding the Pathophysiology of Ischemic Stroke: The Basis of Current Therapies and Opportunity for New Ones. Biomolecules.

[6] Bulygin K.V., Beeraka N.M., Saitgareeva A.R., Nikolenko V.N., Gareev I., Beylerli O., Akhmadeeva L.R., Mikhaleva L.M., Solis L.F.T., Herrera A.S. (2020). Can miRNAs Be Considered as Diagnostic and Therapeutic Molecules in Ischemic Stroke Pathogenesis?—Current Status. Int. J. Mol. Sci..

[7] Cepparulo P., Cuomo O., Vinciguerra A., Torelli M., Annunziato L., Pignataro G. (2021). Hemorrhagic Stroke Induces a Time-Dependent Upregulation of miR-150-5p and miR-181b-5p in the Bloodstream. Front. Neurol..

[8] Braicu C., Catana C., Calin G.A., Berindan-Neagoe I. (2014). NCRNA combined therapy as future treatment option for cancer. Curr. Pharm. Des..

[9] Strmsek Z., Kunej T. (2015). MicroRNA Silencing by DNA Methylation in Human Cancer: A Literature Analysis. Non-Coding RNA.

[10] Calin G.A., Croce C.M. (2006). Genomics of chronic lymphocytic leukemia microRNAs as new players with clinical significance. Semin. Oncol..

[11] Redis R.S., Berindan-Neagoe I., Pop V.I., Calin G.A. (2012). Non-coding RNAs as theranostics in human cancers. J. Cell Biochem..

[12] Pan J.-Y., Sun C.-C., Bi Z.-Y., Chen Z.-L., Li S.-J., Li Q.-Q., Wang Y.-X., Bi Y.-Y., Li D.-J. (2017). miR-206/133b Cluster: A Weapon against Lung Cancer?. Mol. Ther. Nucleic Acids.

[13] Volinia S., Calin G.A., Liu C.G., Ambs S., Cimmino A., Petrocca F., Visone R., Iorio M., Roldo C., Ferracin M. (2006). A microRNA expression signature of human solid tumors defines cancer gene targets. Proc. Natl. Acad. Sci. USA.

[14] Cipolla G., de Oliveira J., Salviano-Silva A., Lobo-Alves S., Lemos D., Oliveira L., Jucoski T.S., Mathias C., Pedroso G.A., Zambalde E.P. (2018). Long Non-Coding RNAs in Multifactorial Diseases: Another Layer of Complexity. Non-Coding RNA.

[15] Braicu C., Cojocneanu-Petric R., Chira S., Truta A., Floares A., Achimas-Cadariu P., Berindan-Neagoe I., Petrut B. (2015). Clinical and pathological implications of miRNA in bladder cancer. Int. J. Nanomed..

[16] Catana C.S., Calin G.A., Berindan-Neagoe I. (2015). Inflamma-miRs in Aging and Breast Cancer: Are They Reliable Players?. Front. Med..

[17] Irimie A.I., Braicu C., Sonea L., Zimta A.A., Cojocneanu-Petric R., Tonchev K., Mehterov N., Diudea D., Buduru S., Berindan-Neagoe I. (2017). A Looking-Glass of Non-coding RNAs in oral cancer. Int. J. Mol. Sci..

[18] Catana C.S., Pichler M., Giannelli G., Mader R.M., Berindan-Neagoe I. (2017). Non-coding RNAs, the Trojan horse in two-way communication between tumor and stroma in colorectal and hepatocellular carcinoma. Oncotarget.

[19] Berindan-Neagoe I., Monroig Pdel C., Pasculli B., Calin G.A. (2014). MicroRNAome genome: A treasure for cancer diagnosis and therapy. CA A Cancer J. Clin..

[20] Braicu C., Calin G.A., Berindan-Neagoe I. (2013). MicroRNAs and cancer therapy—From bystanders to major players. Curr. Med. Chem..

[21] Eastlack S.C., Alahari S.K. (2015). MicroRNA and Breast Cancer: Understanding Pathogenesis, Improving Management. Non-Coding RNA.

[22] Munker R., Calin G.A. (2011). MicroRNA profiling in cancer. Clin. Sci. (Lond. Engl. 1979).

[23] Pop-Bica C., Pintea S., Cojocneanu-Petric R., Del Sal G., Piazza S., Wu Z.H., Alencar A.J., Lossos I.S., Berindan-Neagoe I., Calin G.A. (2018). MiR-181 family-specific behavior in different cancers: A meta-analysis view. Cancer Metastasis Rev..

[24] Gulei D., Petrut B., Tigu A.B., Onaciu A., Fischer-Fodor E., Atanasov A.G., Ionescu C., Berindan-Neagoe I. (2018). Exosomes at a glance—Common nominators for cancer hallmarks and novel diagnosis tools. Crit. Rev. Biochem. Mol. Biol..

[25] Bell-Hensley A., Das S., McAlinden A. (2023). The miR-181 family: Wide-ranging pathophysiological effects on cell fate and function. J. Cell Physiol..

[26] Indrieri A., Carrella S., Carotenuto P., Banfi S., Franco B. (2020). The Pervasive Role of the miR-181 Family in Development, Neurodegeneration, and Cancer. Int. J. Mol. Sci..

[27] Lv B., He S., Li P., Jiang S., Li D., Lin J., Feinberg M.W. (2024). MicroRNA-181 in cardiovascular disease: Emerging biomarkers and therapeutic targets. FASEB J..

[28] An T.H., He Q.W., Xia Y.P., Chen S.C., Baral S., Mao L., Jin H.-J., Li Y.-N., Wang M.-D., Chen J.-G. (2016). MiR-181b Antagonizes Atherosclerotic Plaque Vulnerability Through Modulating Macrophage Polarization by Directly Targeting Notch1. Mol. Neurobiol..

[29] Sun X., Sit A., Feinberg M.W. (2014). Role of miR-181 family in regulating vascular inflammation and immunity. Trends Cardiovasc. Med..

[30] Braicu C., Gulei D., Cojocneanu R., Raduly L., Jurj A., Knutsen E., Calin G.A., Berindan-Neagoe I. (2019). miR-181a/b therapy in lung cancer: Reality or myth?. Mol. Oncol..

[31] Braicu C., Gulei D., Raduly L., Harangus A., Rusu A., Berindan-Neagoe I. (2019). Altered expression of miR-181 affects cell fate and targets drug resistance-related mechanisms. Mol. Asp. Med..

[32] Rezaei T., Amini M., Hashemi Z.S., Mansoori B., Rezaei S., Karami H., Mosafer J., Mokhtarzadeh A., Baradaran B. (2020). microRNA-181 serves as a dual-role regulator in the development of human cancers. Free Radic. Biol. Med..

[33] Han X., Zheng Z., Wang C., Wang L. (2018). Association between MEG3/miR-181b polymorphisms and risk of ischemic stroke. Lipids Health Dis..

[34] Antony M., Scranton V., Srivastava P., Verma R. (2020). Micro RNA 181c-5p: A promising target for post-stroke recovery in socially isolated mice. Neurosci. Lett..

[35] Edwardson M.A., Shivapurkar N., Li J., Khan M., Smith J., Giannetti M.L., Fan R., Dromerick A.W. (2023). Expansion of plasma MicroRNAs over the first month following human stroke. J. Cereb. Blood Flow. Metab..

[36] Bellaousov S., Reuter J.S., Seetin M.G., Mathews D.H. (2013). RNAstructure: Web servers for RNA secondary structure prediction and analysis. Nucleic Acids Res..

[37] Li S., Zhu P., Wang Y., Huang S., Wu Z., He J., Hu X., Wang Y., Yuan Y., Zhao B. (2023). miR-181a targets PTEN to mediate the neuronal injury caused by oxygen-glucose deprivation and reoxygenation. Metab. Brain Dis..

[38] Anatomica LMTTHY-yWY-sAbtpaelotfm-asoisJA. https://jpxb.bjmu.edu.cn/EN/10.16098/j.issn.0529-1356-2022.04.010.

[39] Ma Q., Zhao H., Tao Z., Wang R., Liu P., Han Z., Ma S., Luo Y., Jia J. (2016). MicroRNA-181c Exacerbates Brain Injury in Acute Ischemic Stroke. Aging Dis..

[40] Song X., Xue Y., Cai H. (2021). Down-Regulation of miR-181a-5p Prevents Cerebral Ischemic Injury by Upregulating En2 and Activating Wnt/β-catenin Pathway. J. Stroke Cerebrovasc. Dis..

[41] Shu J., Yang L., Wei W., Zhang L. (2022). Identification of programmed cell death-related gene signature and associated regulatory axis in cerebral ischemia/reperfusion injury. Front. Genet..

[42] Ouyang Y.B., Lu Y., Yue S., Xu L.J., Xiong X.X., White R.E., Sun X., Giffard R.G. (2012). miR-181 regulates GRP78 and influences outcome from cerebral ischemia in vitro and in vivo. Neurobiol. Dis..

[43] Li S., Chen S., Wang Y., Hu X., Wang Y., Wu Z., Huang S., He J., Deng F., Zhao B. (2023). Direct targeting of DOCK4 by miRNA-181d in oxygen-glucose deprivation/reoxygenation-mediated neuronal injury. Lipids Health Dis..

[44] Yang X., Yan S., Wang P., Wang G. (2022). Identification of Hub Genes in the Pathogenesis of Ischemic Stroke Based on Bioinformatics Analysis. J. Korean Neurosurg. Soc..

[45] Hutchison E.R., Kawamoto E.M., Taub D.D., Lal A., Abdelmohsen K., Zhang Y., Wood W.H., Lehrmann E., Camandola S., Becker K.G. (2013). Involvement of miR-181 in Neuroinflammatory Responses of Astrocytes. Glia.

[46] Xue L.X., Shu L.Y., Wang H.M., Lu K.L., Huang L.G., Xiang J.Y., Geng Z., Zhao Y.-W., Chen H. (2023). miR-181b promotes angiogenesis and neurological function recovery after ischemic stroke. Neural Regen. Res..

[47] Zhang L., Li Y.J., Wu X.Y., Hong Z., Wei W.S. (2015). MicroRNA-181c negatively regulates the inflammatory response in oxygen-glucose-deprived microglia by targeting Toll-like receptor 4. J. Neurochem..

[48] Cao D.-W., Liu M.-M., Duan R., Tao Y.-F., Zhou J.-S., Fang W.-R., Zhu J.-R., Niu L., Sun J.-G. (2020). The lncRNA Malat1 functions as a ceRNA to contribute to berberine-mediated inhibition of HMGB1 by sponging miR-181c-5p in poststroke inflammation. Acta Pharmacol. Sin..

[49] Xie W., Li M., Xu N., Lv Q., Huang N., He J., Zhang Y. (2013). MiR-181a regulates inflammation responses in monocytes and macrophages. PLoS ONE.

[50] Zhang L., Dong L.-Y., Li Y.-J., Hong Z., Wei W.-S. (2012). The microRNA miR-181c controls microglia-mediated neuronal apoptosis by suppressing tumor necrosis factor. J. Neuroinflamm..

[51] Walsh K.B., Zimmerman K.D., Zhang X., Demel S.L., Luo Y., Langefeld C.D., Wohleb E., Schulert G., Woo D., Adeoye O. (2021). miR-181a Mediates Inflammatory Gene Expression After Intracerebral Hemorrhage: An Integrated Analysis of miRNA-seq and mRNA-seq in a Swine ICH Model. J. Mol. Neurosci..

[52] Zhuo Y., Chen W., Li W., Huang Y., Duan D., Ge L., He J., Liu J., Hu Z., Lu M. (2021). Ischemic-hypoxic preconditioning enhances the mitochondrial function recovery of transplanted olfactory mucosa mesenchymal stem cells via miR-181a signaling in ischemic stroke. Aging.

[53] Moon J.M., Xu L., Giffard R.G. (2013). Inhibition of microRNA-181 reduces forebrain ischemia-induced neuronal loss. J. Cereb. Blood Flow. Metab..

[54] Yang Y., Cai Y., Zhang Y., Liu J., Xu Z. (2018). Exosomes Secreted by Adipose-Derived Stem Cells Contribute to Angiogenesis of Brain Microvascular Endothelial Cells Following Oxygen-Glucose Deprivation In Vitro Through MicroRNA-181b/TRPM7 Axis. J. Mol. Neurosci..

[55] Xu G., Song X., Wang X., Xue R., Yan X., Qin L., Chang X., Gao J., Chen Z., Song G. (2024). Combined miR-181a-5p and Ag Nanoparticles are Effective Against Oral Cancer in a Mouse Model. Int. J. Nanomed..

[56] Xu L.J., Ouyang Y.B., Xiong X., Stary C.M., Giffard R.G. (2015). Post-stroke treatment with miR-181 antagomir reduces injury and improves long-term behavioral recovery in mice after focal cerebral ischemia. Exp. Neurol..

[57] Griffiths B.B., Ouyang Y.-B., Xu L., Sun X., Giffard R.G., Stary C.M. (2019). Postinjury Inhibition of miR-181a Promotes Restoration of Hippocampal CA1 Neurons After Transient Forebrain Ischemia in Rats. eneuro.

[58] Deng B., Bai F., Zhou H., Zhou D., Ma Z., Xiong L., Wang Q. (2016). Electroacupuncture enhances rehabilitation through miR-181b targeting PirB after ischemic stroke. Sci. Rep..

[59] Kim M., Lee Y., Lee M. (2021). Hypoxia-specific anti-RAGE exosomes for nose-to-brain delivery of anti-miR-181a oligonucleotide in an ischemic stroke model. Nanoscale.

[60] Zhang X., Liu Z., Shu Q., Yuan S., Xing Z., Song J. (2019). LncRNA SNHG6 functions as a ceRNA to regulate neuronal cell apoptosis by modulating miR-181c-5p/BIM signalling in ischaemic stroke. J. Cell Mol. Med..

